# Mapping the Influence of Commercial Detailing on Prescribing Decisions: A Scoping Review

**DOI:** 10.7759/cureus.111349

**Published:** 2026-06-23

**Authors:** Bhadraj R, Krishnabhaskar Mangalasserri

**Affiliations:** 1 Department of Marketing, SCMS Cochin School of Business, Kochi, IND

**Keywords:** academic detailing, commercial detailing, pharmaceutical detailing, physician decision-making, prescribing behaviour, scoping review

## Abstract

Pharmaceutical detailing serves as a primary knowledge-translation strategy, yet a fundamental tension exists between brand-focused commercial detailing and evidence-based academic detailing. In high-velocity clinical environments where patient consultation time often averages less than 10 minutes, pharmaceutical sales representatives (PSRs) function as critical informational conduits. However, the qualitative effectiveness and operational mechanisms of these interactions remain insufficiently mapped in current literature.

This scoping review aims to systematically map the evidence on pharmaceutical detailing, investigate its influence on prescribing behaviour, and identify the theoretical frameworks and variables that drive clinical decision-making.

A six-stage scoping review in accordance with the Preferred Reporting Items for Systematic reviews and Meta-Analyses - Scoping Review (PRISMA-ScR) guidelines to ensure methodological rigor. We searched PubMed, ProQuest, Google Scholar, and Dimensions for English-language studies published between January 2014 and August 2025 using keywords such as physician detailing, academic detailing, educational outreach visits, doctor, physician, prescriber, general practitioner, and prescription decision. We registered the study protocol with the Open Science Framework (OSF) at https://osf.io/re8yz. Studies involving practicing physicians and non-medical prescribers, including both commercial and academic detailing interventions, were included. Data were charted using the Joanna Briggs Institute (JBI) extraction instrument, and study quality was assessed using JBI critical appraisal tools.

From 5,742 records, 23 studies met the inclusion criteria across high- and middle-income healthcare systems. The evidence shows that pharmaceutical detailing increases the likelihood of initiating branded prescriptions, primarily through targeted informational exposure and repeated interactions. Its impact varies across prescriber segments, with differences observed by specialty, experience, and prescribing volume. Studies indicate that scientifically oriented engagement generates stronger responses in specialised care settings. Structural conditions, including high patient volumes and limited opportunity for independent evaluation, increase dependence on externally provided drug information. Regulatory restrictions consistently reduce brand-specific prescribing, while digital detailing improves access and frequency of engagement. Despite these shifts, direct interaction with PSRs remains central to influencing prescribing behaviour.

Pharmaceutical detailing operates as a system-level communication process that integrates informational delivery with behavioural influence within clinical practice. It extends beyond promotion by actively shaping how doctors access, interpret, and apply therapeutic information in prescribing decisions. The balance between informational value and promotional bias depends on the strength of regulatory oversight and the quality of engagement. As communication channels evolve, detailing must maintain credibility and align with evidence-based practice to sustain its effectiveness. Strengthening governance mechanisms and integrating complementary educational approaches will ensure that detailing supports rational, patient-centered prescribing.

## Introduction and background

In the landscape of pharmaceutical marketing, doctors serve as the primary decision-makers, making them the primary target of promotional strategies. Effective patient management necessitates that doctors access accurate, contemporary drug information. To facilitate this, pharmaceutical firms employ pharmaceutical sales representatives (PSRs), or "detailers," to deliver critical clinical data through face-to-face meetings known as pharmaceutical detailing. Although the roots of detailing trace back to the 19th century, the 1940s marked the emergence of the modern paradigm, leading to its widespread adoption across the global pharmaceutical industry [1]. From a behavioural perspective, this study defines commercial detailing as a profit-driven promotional interaction where PSRs deliver targeted clinical and brand information to healthcare professionals [2]. This methodology is utilised to induce the prescription, supply, or use of specific proprietary medications through the distribution of sales materials and samples [3]. Conversely, academic detailing (AD) - delivered by non-profit or academic teams - emerged as a service-oriented intervention to provide unbiased clinical content and expert reviews to counter commercial influence [4], specifically focusing on improving prescribing quality through evidence-based outreach [1,4].

The fundamental objective of detailing empowers doctors to make better-informed therapeutic choices. Within an economic framework, this study operationalizes influence as the measurable change in a physician's prescribing behaviour resulting from detailing exposure. Datta A et al. [5] found that detailing visits and drug samples account for 83% of all prescription promotion directed toward doctors. Consequently, commercial detailing remains the dominant strategy for pharmaceutical companies seeking to shift prescribing habits. This interaction exerts influence by increasing the probability that a clinician initiates a branded prescription for the first time, and by leveraging socio-psychological triggers like reciprocity to increase the prescribing frequency of the promoted drugs [5,6]. Traditionally, this occurs through personal meetings; however, modern practitioners face significant time constraints and heavy patient loads. To address these hurdles, detailing has evolved to include "e-detailing," a technology encompassing interactive, self-service virtual presentations and video-based sessions where PSRs tailor content to specific doctor interests [7].

Recent data underscores the "high-velocity" nature of these clinical environments. Singhal et al. [8] observed that average surgical patient consultations last only 9.8 minutes, while general physician (GP) consultations drop to 2.2 minutes. Furthermore, GPs may see more than 200 patients per day [9]. In this context, the effectiveness of a detailing interaction is paramount. We conceptualize effectiveness as a multi-dimensional construct capturing the quantitative success in prescription volume and the qualitative success regarding prescriber satisfaction and the perceived clinical utility of the information [10,11]. Moreover, effectiveness is evaluated by the accuracy of the clinical knowledge transferred and its alignment with evidence-based guidelines [12,13].

In this context, a PSR must create a lasting impression within seconds to ensure their brand remains top of the mind when the doctor reaches a diagnosis. This cognitive presence is vital for the pharmaceutical company to maintain its market share. With the global industry projected to reach $2.25 trillion by 2028 and the Indian market expanding rapidly, the pressure for effective engagement has never been higher [14]. Indeed, the National Pharmaceutical Pricing Authority (NPPA), India, reports that over 60,000 generic brands compete for attention, necessitating precise product knowledge and selection [15]. As Indian families allocate over 60% of their healthcare budgets to primary care [8], the precision of commercial detailing remains a critical factor in the healthcare ecosystem.

Study rationale and objectives

While detailing represents a significant information transfer strategy, the literature lacks consensus on communication models that balance commercial goals with clinical needs. Most existing studies focus on AD, leaving a gap in understanding the optimal effectiveness of commercial detailing. Furthermore, current metrics often prioritise sales increases over the qualitative effectiveness of PSR interactions and other relevant parameters. Therefore, a scoping review is essential to map the evidence on commercial detailing and systematically analyse this complex landscape.

This review investigates how detailing influences prescribing behaviour by examining how existing studies define and delineate the scope of pharmaceutical detailing, how detailing affects doctors, patients, and pharmaceutical companies, and which theoretical frameworks explain the operational mechanisms of detailing and guide best practices.

## Review

Methods

We conducted this scoping review to map the multifaceted influence of commercial detailing on prescription behaviour. The complex and heterogeneous nature of industry-physician interactions, including variability in study designs, outcome measures, and contextual settings, made a scoping review methodology more appropriate than a systematic review focused on effect estimation. We emphasize that this approach aims to identify key patterns of association, underlying mechanisms, and research gaps rather than to establish causal relationships.

We registered the study protocol on the Open Science Framework (OSF) registries (https://osf.io/re8yz). We conducted the review in accordance with the six-stage methodological framework proposed [9], incorporating refinements [10] and guidance from the Joanna Briggs Institute (JBI) manual for evidence synthesis. To ensure transparency and methodological rigor, we adhered to the Preferred Reporting Items for Systematic Reviews and Meta-Analyses Extension for Scoping Reviews (PRISMA-ScR) checklist [11].

Stage 1: Identifying the Research Question

The primary research question investigates how pharmaceutical detailing influences the prescribing behaviour of doctors.

Stage 2: Identifying Relevant Literature

Data sources and search strategy: We developed a comprehensive search strategy through a preliminary literature scan, internal research discussions, and consultations with pharmaceutical industry experts. We searched four primary databases: PubMed, ProQuest, Google Scholar, and Dimensions, focusing on the period between January 2014 and August 2025. We combined Population (P), Concept (C), and Context (C) terms using Boolean operators, with key search terms including physician detailing, academic detailing, educational outreach visits, doctor, physician, prescriber, general practitioner, and prescription decision (Appendix 1).

Stage 3: Selection of Sources of Evidence

We ensured methodological rigor through a team-based consensus model. We calibrated the screening protocol by independently testing 50 citations, which allowed us to refine the operational definitions of our PCC (Population-Concept-Context) framework. Both authors independently screened all records to eliminate selection bias. We resolved conflicts through thematic discussion, ensuring that final inclusion required unanimous agreement. To ensure transparency, we maintained a verifiable audit trail using a shared data charting form that documented every decision-making step from initial identification to final inclusion. The review included practicing doctors and non-medical prescribers exposed to commercial (promotional visits, samples, gifts) or AD (evidence-based outreach), with outcomes related to prescribing behaviour or stakeholder impact across all healthcare settings. The eligible sources comprised quantitative, qualitative, and mixed-methods studies, along with relevant reviews, industry reports, and policy documents published in English between January 2014 and August 2025. The review excluded studies without a personal detailing component, non-prescriber populations, non-English publications, and abstract-only or pre-2014 records.

Stage 4: Data Extraction and Quality Assessment

We charted data using the JBI extraction instrument to create a descriptive summary of the evidence [16]. Two authors independently screened studies based on titles and abstracts for inclusion. Subsequently, both reviewers independently reviewed the full-text manuscripts and extracted key data while also assessing the methodological quality of each included study. The authors resolved disagreements through discussion until they reached consensus.

The review extracted and summarized key study characteristics, including authors, publication year, country, study period, study design, objectives, study population, sample size, and reported outcomes. In addition to descriptive extraction, we systematically mapped JBI appraisal outcomes to each included study to support a quality-informed thematic synthesis. This approach enabled us to contextualize findings according to methodological rigor without applying quantitative weighting, thereby maintaining consistency with scoping review methodology. As a scoping review, this study does not aim to establish causality or estimate effect size, but rather to map evidence and identify patterns across heterogeneous studies. Study screening and data management utilized Microsoft Excel (Redmond, WA, USA), which facilitated independent review and conflict tracking. We pilot-tested the data charting form on a subset of included studies and refined it iteratively to ensure completeness and consistency across reviewers. We charted the extracted data using the JBI extraction instrument to generate a structured descriptive summary of the evidence (Table 1) [16].

**Table 1:** Characteristics of Included Studies This table includes studies with extractable empirical characteristics (n=21) and excludes two policy documents [14,15] due to the absence of standardized methodological data; however, these documents inform contextual interpretation and strengthen analytical depth. PSRs: pharmaceutical sales representatives, AD: academic detailing, MRs: medical representatives

Author Name & Year of Publication	Study Design	Objective	Outcomes	Quality Assessment
Francer et al., 2014 [3]	Narrative review; Country and participants: global, not applicable	Explores the evolution of international codes and national regulations that ensure the quality of promotional or scientific materials distributed to healthcare stakeholders	Provides extensive global and regional regulatory codes that define how and what scenario promotional detailing as advertising- and sales-based inputs are distributed to healthcare professionals via PSR	High
Yeh et al. (2016) [1]	Consensus development study (Delphi Method); Country: USA; Participants: 20 experts (researchers and experienced detailers)	To establish a formal expert consensus on the essential components and best practices for implementing AD programs to ensure consistency and improve evaluation standards	Expert consensus was established for 46 core indicators across seven thematic domains: program environment, clinical prioritization, educational collateral, detailer competency, encounter dynamics, physician engagement, and evaluative metrics	High
Datta and Dave (2017) [5]	Quantitative, cross-sectional study; Country: USA; Participants: 149,247 doctors	To what extent does pharmaceutical detailing increase the number of new prescriptions written for a specific branded drug	Detailing increases extensive margin to prescribe a drug for first time and ensures selective demand allows pharma firms to gain market share from competitors	High
Workneh et al. (2016) [6]	Quantitative, descriptive cross-sectional study; Country: Ethiopia; Participants: 114 doctors	Evaluates the extent to which PSRs influence the prescribing habits of physicians	48.2% of doctors were influenced by PSR visits and detailing	High
Brax et al. (2017) [12]	Systematic review and meta-analysis; Country and participants: global, not applicable	To review the association between physicians' interactions with pharmaceutical companies and their clinical practice	Detailing visits lead to a significant increase in prescribing frequency for the promoted products	High
Murshid and Mohaidin (2017) [17]	Systematic review; Country and participants: global, not applicable	To address the theoretical deficit in pharmaceutical marketing research by reviewing existing prescribing models	Detailing acts as a marketing stimulus that targets doctors' cognitive and emotional processing. It scopes it as a component of environmental factors that compete with patient needs and professional norms	High
Larkin et al. (2017) [18]	Quantitative study; Country: USA; Participants: 2,126 doctors	To analyse the association between detailing policies enacted at Academic Medical Center (AMCs) and physician prescribing of actively detailed and not detailed drugs	Restriction in pharmaceutical detailing was associated with a significant reduction in prescribing detailed drugs	High
Fickweiler et al. (2017) [13]	Systematic review; Country and participants: global, not applicable	To analyse the nature and interactions between doctors and PSRs	PSRs are perceived as important sources of pharmaceutical information to doctors	High
Balkanski and Getov (2019) [7]	Textual evidence; Country and participants: not applicable	To provide a comprehensive framework for pharmaceutical companies to transition from traditional face-to-face sales models to integrated digital marketing strategies	Commercial detailing operates best through a hybrid model, where digital tools act as a "force multiplier" with PSRs (traditional face-to-face detailing) rather than a replacement	Moderate
Schwartz and Woloshin (2019) [19]	Longitudinal review; Country and participants: global, not applicable	To quantify expenditures and trends in pharmaceutical promotion to health professionals (detailing) and consumers, while evaluating the resulting clinical and regulatory consequences	Face-to-face detailing functions as the central node of pharmaceutical promotion, initiating secondary marketing channels and maximizing physician recall through high-engagement personal interaction	High
Pandey et al. (2019) [9]	Quantitative, cross-sectional observational study; Country: India; Participants: not applicable	To measure the patient-to-doctor ratio and the time allocated per patient in super-specialty outpatient departments (OPDs)	Mean consultation times varied significantly by department: medical sub-specialties (Gastroenterology/Neurology/Oncology) averaged 2.0 minutes per patient amidst high volumes (>200/day), compared to 2.2 minutes for super-specialties and 10.0 minutes for surgical disciplines	High
Smart et al. (2020) [11]	Exploratory factor analysis; Country: USA; Participants: not mentioned	To develop an instrument to capture the detailer’s perception of the effectiveness of AD	Psychometric evaluation of the six-item scale confirmed strong reliability (0.79-0.82) and factor validity. The instrument effectively operationalizes perceived effectiveness as a single latent construct, facilitating standardized assessment of AD interventions	Low
Altawalbeh et al. (2020) [20]	Quantitative, descriptive and analytical cross-sectional study; Country: Jordan; Participants: 310 doctors	To examine the influence of commercial detailing on prescribing behavior among private practitioners in Jordan and measure professional readiness to adopt academic	73% doctors agreed that pharmaceutical promotion influences their practices. 66% of doctors agreed that pharma-sponsored lectures are biased	High
Jandhyala (2020) [21]	Quantitative, cross-sectional pilot survey; Country: UK; Participants: 122 prescribers (physicians, nurses, and pharmacists)	Evaluates the impact of non-personal and non-promotional engagement on the prescribing patterns of UK healthcare professionals, utilizing traditional face-to-face promotional detailing (comparator)	Non-promotional personal engagement with doctors and PSRs had the highest influence on prescription	High
Price et al. (2021) [22]	Quantitative, cross-sectional survey; Country: USA; Participants: 122 prescribers (physicians, nurses, and pharmacists)	To identify the primary factors shaping the prescribing decisions of healthcare providers in the United States	53% of healthcare professionals engage with PSR monthly, even though clinical guidelines and professional peers are ranked as the most significant drivers of clinical decision-making	High
Boudewyns et al. (2021) [23]	Quantitative, experimental study; Country: USA; Participants: 1,311 oncologists	To investigate how oncologists perceive the clinical utility of preliminary or exploratory data when presented in promotional materials	Detailing exerts influence through 'cognitive framing' that persists despite regulatory disclosures	High
Monteiro et al. (2022) [10]	Methodological research (validation study); Country: USA; Participants: 183 doctors	To develop and validate the Prescriber Satisfaction with Academic Detailing (PSAD) instrument to provide a reliable way to measure healthcare provider satisfaction following an AD encounter	Cognitive biases and time constraints frequently prevent healthcare providers from validating pharmaceutical claims, leading to a disproportionate reliance on expert-driven persuasion and industry-curated information by PSRs	High
Shaarani et al. (2024) [2]	Quantitative, descriptive cross-sectional study; Country: Lebanon; Participants: 268 doctors	To evaluate the attitudes, beliefs, and behaviours of Lebanese doctors concerning their interactions with PSRs	The synergy between detailing and low-value incentives triggers a social exchange mechanism; this turn triggers obligation to reciprocate, shifting prescribing habits toward promoted brands through non-clinical drivers	High
Singhal et al. (2024) [8]	Quantitative, descriptive cross-sectional study; Country: India; Participants: 268 doctors	To evaluate the current status of communication practices among Indian clinicians	Clinical consultations average a mere 9.8 minutes, characterized by significant communication gaps: while 82.8% of doctors report attentive listening, 80% omit formal introductions, and nearly half fail to verify patient comprehension, directly undermining medication adherence	Moderate
Uniform Code for Pharmaceutical Marketing Practices (UCPMP) 2024 - Reg 2024 [24]	Regulatory policy; Country: India; Participants: not applicable	Guideline for ethical boundaries for pharmaceutical "detailing" and promotion in the Indian market	Defines detailing (promotion) as any activity intended to "induce the prescription, supply, or use of medical drugs." It asserts that MRs must not use inducements to gain access to doctors	High
Rome et al. (2025) [4]	Systematic review; Country and participants: global, not applicable	To evaluate the effectiveness of AD on prescription behaviour of doctors	Conceptualizes detailing as a distinct behavioral change instrument leveraging interpersonal dynamics and describes commercial detailing as profit-driven and AD as evidence-driven	High

Simultaneously, we evaluated study quality using the JBI critical appraisal checklist [16]. The assessment evaluated methodological rigor across key domains, including clarity of research objectives, appropriateness of study design, sampling strategy, validity and reliability of measurements, management of confounding factors, analytical rigor, and transparency in reporting results and limitations.

The methodological transparency was enhanced by refining the description of the search strategy, study selection process, and data charting procedures. We clarified the application of the Arksey and O’Malley framework with Levac et al. refinements and explicitly aligned each stage with the PRISMA-ScR reporting standards [25,26]. We also improved reproducibility by detailing database-specific search logic, inclusion-exclusion criteria, and the screening workflow that reduced 5,742 records to 23 included studies.

Weighted interpretation of evidence: The synthesis prioritised high-quality studies to support key conclusions, with strong associations between detailing frequency and prescribing behaviour derived from robust quantitative and systematic evidence [1,4]. High-quality studies (87%) consistently demonstrated that detailing increases branded prescribing, while policy restrictions in Academic Medical Centres reduce such prescribing, indicating a causal link between promotional access and clinical decisions [18].

Contextual use of lower-quality evidence: Moderate-quality evidence, particularly on digital and hybrid detailing, was interpreted cautiously as indicative of emerging trends rather than definitive findings [7]. Low-quality studies informed conceptual gaps without supporting inferential claims.

Integration into synthesis and theory: The review embedded quality appraisal within thematic analysis, relying on high-quality studies to support behavioural mechanisms such as reciprocity [6], while acknowledging potential biases. Theoretical frameworks, including the Elaboration Likelihood Model (ELM) and Theory of Planned Behavior (TPB), structured the interpretation of findings [17].

The quality appraisal informed the analytical hierarchy rather than study exclusion, ensuring that conclusions reflect both the strength and limitations of the evidence base.

Stage 5: Results

**Figure 1:**
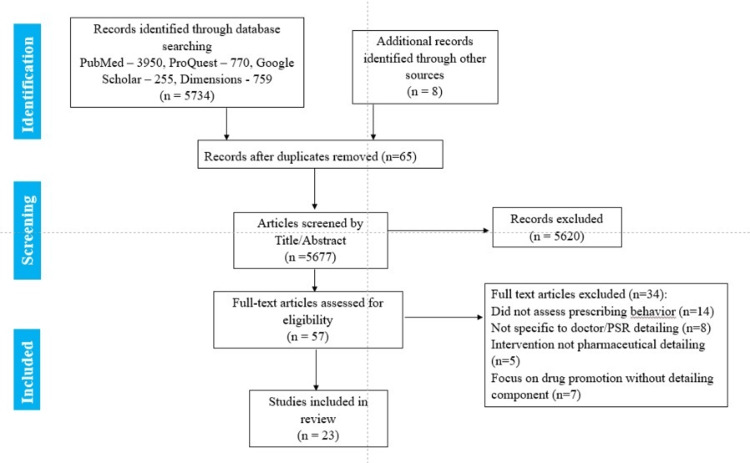
Preferred Reporting Items for Systematic Reviews and Meta-Analyses Extension for Scoping Reviews (PRISMA-ScR) Flow Diagram for Scoping Review PSR: pharmaceutical sales representative

We used the PRISMA-ScR flow diagram to document and report the study selection process (Figure 1).

Results

The systematic search identified 5,734 records across PubMed, ProQuest, Google Scholar, and Dimensions. An additional eight studies were identified through manual searching of cited references, resulting in 5,742 records. After removing 65 duplicates, 5,677 unique titles and abstracts underwent screening. Of these, 5,623 records were excluded during the initial screening phase due to insufficient alignment with the review scope, and 57 articles proceeded to full-text evaluation. Following full-text assessment, 34 papers were excluded for not specifically addressing detailing’s influence on prescribing behaviour. Full-text exclusions occurred for the following reasons: did not assess prescribing behavior (n=14), not specific to doctor/PSR detailing (n=8), intervention not pharmaceutical detailing (n=5), and focus on drug promotion without a detailing component (n=7). A total of 23 studies met the inclusion criteria and were included in the final analysis.

The included literature covers a broad geographical spectrum. The United States contributed seven studies, followed by India (three), Ethiopia (one), Jordan (one), Lebanon (one), and the United Kingdom (one). Six review papers provided a global perspective. Table 1 summarizes the key characteristics of the studies included in this review.

The review categorized included studies based on methodological design, including observational (cross-sectional and longitudinal), experimental or quasi-experimental, narrative reviews, and policy or regulatory analyses. The synthesis followed a design-stratified approach, where the analysis first examined findings within each study category and subsequently integrated them at a conceptual level. Empirical studies informed observed patterns of association, while narrative and policy sources contributed to theoretical and contextual interpretation. This structured approach prevented inappropriate aggregation of heterogeneous evidence and enhanced the validity and transparency of the synthesis.

A proportion of included studies rely on physician self-reported data, which introduces susceptibility to social desirability and recall bias. Physicians may underestimate the influence of pharmaceutical promotion due to an “illusion of invulnerability,” perceiving themselves as less affected than their peers. Consequently, self-reported findings reflect perception-based outcomes rather than directly observed prescribing behaviour. The analysis therefore treats these findings as analytically distinct from evidence derived from observational or longitudinal designs. This distinction preserves methodological rigor, prevents over-interpretation of subjective data, and ensures that the synthesis remains aligned with the strengths and limitations of the underlying evidence.

Commercial detailing exerts a measurable and structured influence on prescribing behaviour, shaping drug selection through the interaction of (1) commercial detailing vs AD, (2) Extensive Margin and Brand Selection, (3) Socio-Psychological Mechanisms: Incentives and Reciprocity, (4) Digital Integration and Hybrid Engagement Models, and (5) Regulatory and Institutional Mediators of Detailing Effectiveness. The evidence demonstrates that these interdependent themes collectively drive prescribing decisions within routine clinical practice rather than isolated promotional interactions.

Commercial Versus Academic Detailing

The literature identifies a clear distinction between commercial and AD in terms of intent, delivery, and impact on prescribing behaviour. Commercial detailing operates as a promotional interaction designed to influence prescribing through targeted engagement by PSRs [2], and is defined within regulatory frameworks such as the Uniform Code for Pharmaceutical Marketing Practices (UCPMP) 2024 as activities intended to induce prescription of specific drugs. In contrast, AD functions as an evidence-based educational intervention delivered by non-commercial experts to support rational prescribing and patient safety [1,4]. Although industry guidelines aim to ensure informational accuracy, evidence suggests that commercial detailing may introduce persuasive bias due to underlying commercial interests, which self-regulation may not fully mitigate [3]. This distinction highlights a structural divergence in objectives, where commercial detailing primarily drives brand-oriented decisions, while AD aligns with evidence-based clinical practice.

Extensive Margin and Brand Selection

Commercial detailing effectively increases a drug’s market share by influencing doctors to initiate new prescriptions, rather than merely expanding the overall patient pool. Datta A et al. [5] demonstrate that detailing increases the "extensive margin," which is the probability of a doctor prescribing a specific branded drug for the first time. This creates "selective demand," allowing firms to capture market share from competitors. Brax et al. [12] support this, finding a significant positive correlation between the frequency of PSR visits and the volume of prescriptions written for promoted products. However, some studies suggest this effect is moderated by the presence of generic alternatives; for example, the availability of a generic can dampen the prescribing response to branded detailing [5]. This underscores that detailing's primary success lies in its ability to dictate specific brand choices within time-constrained clinical environments.

Socio-Psychological Mechanisms: Incentives and Reciprocity

Detailing interactions activate socio-psychological mechanisms, particularly reciprocity, which generate implicit obligations that influence prescribing decisions. Commercial detailing shapes prescribing behaviour by leveraging drug samples and low-value promotional incentives to facilitate initial brand adoption. Workneh et al. [6] reported that 48.2% of physicians in Ethiopia acknowledged the direct impact of such interactions on their prescribing patterns. Empirical evidence further indicates that increased frequency of representative visits and greater availability of free samples significantly enhance the likelihood of physicians initiating prescriptions for specific branded medications [5]. Brax et al. [12] corroborate these findings, demonstrating a positive association between repeated interactions and higher prescribing volumes, alongside a preference for branded drugs over generics. However, the influence of these incentives remains context-dependent, as the presence of generic alternatives can attenuate the impact of branded promotion [5]. Shaarani et al. [2] highlight the role of social exchange dynamics, where low-value incentives such as meals or stationery reinforce reciprocal behaviour and shift prescribing toward promoted brands. Notably, a perceptual divergence persists, with physicians often attributing susceptibility to peers while maintaining self-perceived objectivity [2]. This gap underscores the role of implicit cognitive bias in sustaining the influence of non-clinical drivers within prescribing behaviour.

Digital Integration and Hybrid Engagement Models

Systemic time constraints within clinical practice significantly increase physicians’ reliance on industry-curated information, a dependence that has intensified with the evolution of traditional, e-detailing, and hybrid engagement models. In high-volume settings such as India, where mean consultation times range between 2.0 and 2.2 minutes, clinicians face limited capacity for independent evidence appraisal during routine practice [8,9]. Consequently, PSRs function as accessible sources of concise, pre-processed clinical information, effectively providing “executive summaries” within constrained decision environments.

The transition toward digital integration expands engagement beyond face-to-face interactions, with traditional detailing remaining the central node of promotion while digital platforms (web and mobile) enhance continuous access to clinical content [7,19]. E-detailing enables asynchronous and scalable information delivery, improving accessibility and operational efficiency. However, this shift does not fundamentally alter the cognitive context of decision-making. Monteiro et al. [10] demonstrate that time pressure and cognitive load continue to drive reliance on expert-curated information rather than independent evaluation.

Hybrid models, which combine interpersonal and digital channels, therefore represent an adaptive evolution rather than a substitution. While digital tools extend reach and persistence, the personal relationship with PSRs remains the primary driver of prescribing intent [17]. This convergence positions detailing as a system-embedded mechanism in contemporary clinical practice, where traditional, digital, and hybrid modalities collectively shape physician behaviour through sustained informational and relational influence.

Regulatory and Institutional Mediators of Detailing Effectiveness

The efficacy of pharmaceutical detailing is not absolute but it is significantly shaped by the external regulatory environment and internal institutional policies that govern doctor-industry interactions. Francer et al. [3] highlight that regulatory control systems, including industry codes and national laws, are fundamental in determining the rationality of prescribing behaviour. Furthermore, Larkin et al. [18] demonstrate a direct causal link between policy and practice, showing that restricting PSR access at Academic Medical Centres (AMCs) leads to a significant reduction in the prescribing of detailed branded drugs. While institutional restrictions prove effective, a critical evaluation suggests that these policies may have unintended consequences. While recent studies argue that banning gifts and samples significantly reduces the market share of expensive detailed drugs [18], others contend that this effect is moderated by the underlying information needs of physicians, who may seek out digital e-detailing channels to fill the educational gap left by restricted physical visits [7]. These findings are vital to our research question, as they identify policy as a primary independent variable that can either amplify or mitigate detailing's influence (Table 2).

**Table 2:** Thematic Summary: Commercial Detailing and Prescribing Behaviour This table presents studies reporting quantifiable prescribing or behavioural outcomes (n=12) PSRs: pharmaceutical sales representatives

Theme	Focus	Key Insight	Implication
Commercial vs Academic Detailing	Promotional vs evidence-based physician engagement	Commercial detailing is brand-driven and may introduce bias, while academic detailing supports rational prescribing [1,3,4]	Clear divide between market-driven influence and evidence-based education
Extensive Margin & Brand Choice	First-time prescribing of branded drugs	Detailing increases likelihood of initiating specific brands; effect reduced by generics [5,12]	Main impact is on brand selection, not total prescription volume
Reciprocity & Incentives	Social and psychological influence	Samples, visits, and incentives trigger reciprocity, increasing branded prescribing [5,6,12]	Prescribing is shaped by implicit behavioral bias and social exchange
Digital & Hybrid Models	Shift to e-detailing and hybrid engagement	Time pressure drives reliance on PSRs; digital tools extend but do not replace influence [8-10,19]	Hybrid models strengthen continuous exposure and influence
Regulatory & Institutional Factors	Policy control over detailing	Restrictions reduce branded prescribing, but substitution via digital channels may occur [7,18]	Regulation moderates impact but does not eliminate influence

Determinants and Pathways of Prescribing Decision

Our review identified several detailing variables (Table 3) that characterise the relationship between detailing and clinical outcomes.

**Table 3:** Detailing Factors Affecting Prescription Behavior This table includes studies contributing to theoretical and conceptual synthesis based on thematic relevance (n=16). PSR: pharmaceutical sales representative, FDA: Food and Drug Administration, AMC: academic medical center, DTC: direct-to-consumer, HCP: healthcare provider, NSAID: nonsteroidal anti-inflammatory drug

Author & Year of Publication	Nature of Intervention	Associated Variable Independent Variables (IV), Dependent Variables (DV)
Francer et al. (2014) [3]	Intervention: Pharmaceutical detailing and promotional communications, including the distribution of sales materials and scientific data by company representatives. Comparator: Self-regulatory code-based systems against statutory legal enforcement across different jurisdictions. Inference: Activities such as sponsorship for medical education, hospitality limits, and the certification of promotional aids are critical in promotion	IV: Regulatory control systems (industry codes, company standards, national laws, and regulations). DV: Prescribing behaviour (rationality and accuracy), patient welfare, and industry compliance levels
Datta and Dave (2017) [5]	Intervention: Pharmaceutical detailing visits and sample distribution. Comparator: The study compares prescribing behaviors across varying intensities of promotion and evaluates how the presence of a generic alternative (acyclovir) moderates the detailing response. Inference: The average detailed doctors received approximately one representative visit every three months	IV: Number of detailing visits by PSR and the volume of free drug samples provided to the physician's office. DV: Total count of new prescriptions (NRX) written per month for the detailed drug (Famvir), substitute branded drugs (Valtrex), and generic alternatives (acyclovir)
Workneh et al. (2016) [6]	Intervention: Exposure to face-to-face pharmaceutical detailing and promotional incentives. Comparator: This study uses internal comparison groups (e.g., physicians with low vs. high frequency of PSR interaction). Inference: Doctors received promotional items ranging from low-cost marketing inputs to high-value incentives	IV: Frequency of PSR visits, types of gifts accepted (stationery, drug samples, meals, financial incentives), and physician seniority/specialty. DV: Perceived and actual influence on prescribing behavior (likelihood of prescribing the promoted drug)
Brax et al. (2017) [12]	Intervention: Exposure to pharmaceutical detailing and industry-related promotional activities. Comparator: Doctors with no exposure or lower levels of exposure to industry detailing. Inference: Varied intensities of detailing, ranging from occasional visits to weekly PSR interactions found to be significant	IV: Frequency of pharmaceutical detailing visits, receipt of promotional gifts (meals, stationery, samples), and attendance at industry-sponsored continuing medical education (CME). DV: Prescribing frequency (volume), prescribing cost (brand-name vs. generic preference), and prescribing quality (adherence to evidence-based guidelines)
Murshid and Mohaidin (2017) [17]	Comparator: This study compares the explanatory power of different theoretical lenses (e.g., Agency Theory vs. Theory of Planned Behavior). Inference: Provided vital facts how detailing acts as a persuasive communication tool that travels through central or peripheral routes of cognition	DV: Doctor prescribing decision-making and behavioural intention. Mediating/Moderating Variables: Habit persistence, perceived risk, and the physician-industry relationship quality
Larkin et al. (2017) [18]	Intervention: Institutional policies restricting salesperson visits to physicians, including bans on gifts, meals, and unsupervised access to clinical areas. Comparator: A matched control group of similar doctors practicing in environments without restrictive detailing policies. Inference: The study analyzed changes over a 78-month period, looking at 10 to 36 months of data post-policy implementation	IV: Implementation of restrictive detailing policies at the AMC level (binary indicator changing over time). DV: The monthly market share percentage of detailed drugs and non-detailed (often generic) drugs within eight therapeutic classes
Fickweiler et al. (2017) [13]	Comparator: Physicians with no industry interaction or baseline behavior before exposure. Inference: Interactions often occurred during working hours, involving the distribution of samples and informational presentations by PSRs	IV: Type and frequency of detailing (e.g., face-to-face meetings, industry-sponsored CME, travel funding, free drug samples, and promotional gifts like meals or stationery). DV: Doctors' attitudes toward industry, clinical knowledge accuracy, prescribing frequency of branded vs. generic drugs, and formulary addition requests
Balkanski and Getov (2019) [7]	Intervention: Implementation of e-detailing programs including self-guided interactive sessions, "e-sampling," and virtual live meetings with sales reps. Comparator: Traditional detailing (physical visits by sales representatives). Inference: Intervention focuses on providing 24/7 access to clinical data, drug indications, and interactive scientific modules	IV: Type of digital channel (Web-based, portal-based, mobile), frequency of digital interaction, and quality of content. DV: Doctor engagement rates, prescription volume, marketing cost-efficiency, and doctor satisfaction
Schwartz and Woloshin (2019) [19]	Intervention: Professional detailing (face-to-face sales visits) and drug sampling. Comparator: Trends in consumer-directed marketing vs. professional-directed marketing over time. Inference: Detailing remains the largest segment of professional marketing	IV: Type of marketing channel (detailing, samples, DTC ads), and regulatory policy changes. DV: Total marketing expenditure, number of promotional ads, frequency of FDA notice of violation letters, and total legal settlements/fines
Altawalbeh et al. (2020) [20]	Intervention: Exposure to traditional pharmaceutical sales representative visits and promotional materials. Comparator: Compares the perceived value of "Commercial Detailing" against the theoretical interest in "Academic Detailing." Inference: Measures the influence of specific promotional tools, such as drug samples, gifts, and sponsored meals	IV: Doctor demographics, frequency of representative visits, and perceived quality of information. DV: Attitudes toward promotion (Acceptance vs. Skepticism) and influence on prescribing behaviour
Jandhyala (2020) [21]	Intervention: Exposure to non-promotional engagement (e.g., medical science liaisons, PSR) and non-personal engagement (e.g., clinical trial data). Comparator: Traditional promotional personal engagement (detailing by PSR). Inference: Compares "peer-to-peer" scientific relationships against traditional commercial "sales" visits	IV: Types of engagement (Promotional Personal, Non-Promotional Personal, Non-Personal). DV: Influence on the decision to prescribe a specific rare disease medication
Price et al. (2021) [22]	Intervention: Natural exposure to various forms of industry promotion (In-person detailing, E-detailing, and sponsored events). Comparator: Comparison of influence levels between different information sources (e.g., scientific journals vs. industry sales reps). Inference: Frequency of PSR visits (daily, weekly, monthly) and the specific types of "perks" received (meals, samples, educational materials) depend on the prescribing potential	IV: HCP professional role, years in practice, and frequency of industry contact. DV: Perceived influence of detailing, frequency of drug sample acceptance, and knowledge of FDA regulations
Boudewyns et al. (2021) [23]	Intervention: Mock pharmaceutical sales aids (detailing materials) containing exploratory data with varying disclosure statements. Comparator: A control group exposed to the same exploratory data but with no disclosures regarding limitations or uncertainty. Inference: Tested specific language, such as Results are exploratory vs. detailed technical explanations of why the data might be unreliable	IV: Exposure to different disclosure types (technical vs. non-technical) and the presence/absence of clinical uncertainty statements. DV: Perceived clinical utility, perceived drug effectiveness, and intention to prescribe
Shaarani et al. (2024) [2]	Intervention: Exposure to PSR visits and promotional gift offerings. Comparator: Comparison of "Self-Perceived Influence" (how much I am influenced) versus "Peer-Perceived Influence" (how much my colleagues are influenced). Inference: Gifts were categorized into low-cost (pens, notebooks), medium-cost (sponsored meals), and high-cost (conference travel, honoraria)	IV: Physician specialty, years in practice, and frequency of contact with PSRs. DV: Acceptance of gifts, belief in the ethicality of gifts, and perceived influence on prescribing
Singhal et al. (2024) [8]	Intervention: Standard clinical practice/observation of current communication habits. Comparator: Comparison between gender groups (male vs. female) and professional behaviors (e.g., listening vs. introducing oneself). Inference: Measured specific behavioral markers like "checking for comprehension" and "active listening"	IV: Gender and type of medical institution (Government vs. Private). DV: Consultation duration, introduction frequency, use of patient names, and empathy levels
Rome et al. (2025) [4]	Intervention: Educational outreach visits by academic detailers (pharmacists, physicians, or nurses) focusing on non-commercial, evidence-based data. Comparator: Usual care or passive educational materials (e.g., mailed guidelines). Details: Interventions varied from single sessions to multi-visit programs, often supplemented by "audit and feedback" reports	IV: Academic detailing interventions (delivery mode, duration, and frequency). DV: Clinical prescribing outcomes (e.g., reduction in opioid/NSAID use, increase in statin or anticoagulant use)

Multilevel theoretical framework of commercial detailing: The multifactorial cognitive and affective determinants of prescription decisions are conceptualized using a theoretical model (Table 4). The framework systematically applies Agency Theory, ELM, and TPB as complementary yet analytically distinct lenses, enabling structured interpretation and critical comparison of mechanisms underlying detailing influence.

**Table 4:** Theoretical Model PSR: pharmaceutical sales representative

Theory/Model	Central Theme	Critical Aspect for Detailing	Relation to Prescription Behavior
Agency Theory	Doctor as an “agent” acts between the patient (principal) and the pharmaceutical company (principal)	Explains conflict of interest, dependency, and trust dynamics	Doctors balance patient welfare and firm influence; detailing exploits information asymmetry
Theory of Planned Behavior (TPB)	Behavior is guided by attitude, subjective norms, and perceived behavioral control	Attitude toward PSRs, social pressure from patients/pharmacists, and control over prescribing	Predicts intention to prescribe promoted drugs
Persuasion Theory / Elaboration Likelihood Model (ELM)	Individuals process persuasive messages via central (rational) or peripheral (emotional) routes	PSRs use emotional appeals, repetition, and relationship building	Doctors' information processing (attention, trust) mediates detailing impact
Stimulus–Response (S-R) / Buyer Behavior Model	Marketing stimuli trigger behavioral responses via the “black box” of cognitive/emotional processing	Marketing mix (4Ps—Product, Price, Place, Promotion)	Doctors' prescribing acts as a purchase decision under influence of marketing stimuli
Social Power Theory	Influence arises from perceived expertise or authority	Pharmacists’ expert power and collaborative influence	Expertise and trust modulate prescribing aligned with pharmacist input

At the core of detailing influence lies a fundamental role conflict, where the doctors must balance their ethical duty as a patient advocate with their position as a high-value consumer for the pharmaceutical industry. Murshid MA et al [17] argue that prescribing is a multifactorial process blending rational logic with emotional triggers. The analysis applies Agency Theory to identify mechanisms of information asymmetry, incentive alignment, and role conflict, where detailing exploits informational advantages and potentially shifts a doctor's focus from patient welfare to industry influence [17]. However, this economic perspective assumes bounded rationality and may underrepresent professional autonomy. In contrast, TPB suggests that prescribing reflects socially mediated intentions rather than purely information-driven distortions, thereby challenging the deterministic implications of Agency Theory. Synthesis of the literature demonstrates that commercial detailing steers decisions away from evidence-based protocols toward promoted options [17], while institutional restrictive policies and the presence of generic alternatives moderate this effect [5,18]. This tension between structural influence and behavioural agency explains why detailing remains both effective and variably resisted across contexts.

Cognitive processing and ELM: The analysis applies the ELM to classify cognitive processing pathways underlying detailing interactions. Pharmaceutical detailing bypasses rational clinical scepticism by leveraging emotional and social cues rather than purely scientific evidence. The ELM model demonstrates that PSRs use repetition and relationship building to activate peripheral processing mechanisms [17]. This process allows persuasive messages to take root without requiring rigorous central evaluation of clinical data. Critics of this model argue that highly specialised clinicians may be more resistant to peripheral cues than generalists. However, recent studies demonstrate that even oncologists remain susceptible to "cognitive framing" in detailing materials, which persists despite regulatory disclosures [23]. The synthesis reconciles these perspectives by demonstrating that detailing operates along a continuum of cognitive engagement rather than exclusively through peripheral processing.

Behavioural intentions and social power dynamics: The analysis applies the TPB to structure findings related to prescribing intentions, while Social Power Theory explains how perceived authority and expertise shape influence. The doctor’s intention to prescribe reflects attitudes toward PSRs and social pressures from peers [17]. The synthesis maps evidence on peer influence, authority perception, and relational engagement to TPB constructs, demonstrating consistent behavioural patterning. The Stimulus-Response (S-R) Model conceptualizes detailing as a direct marketing stimulus (Product, Price, Place, Promotion) that triggers behavioural responses; however, the analysis demonstrates that outcomes do not follow a purely linear pathway. Some studies identify monthly engagement with PSRs as a primary driver of prescriptions [23], whereas others demonstrate that relationship quality and perceived risk moderate this effect [18]. This synthesis demonstrates that prescribing behaviour emerges from the interaction between structured marketing stimuli and socially mediated behavioural intentions.

The literature presents a consistent pattern regarding the efficacy of detailing while identifying key moderating factors. The analysis positions face-to-face detailing as a central node of pharmaceutical promotion, initiating secondary marketing channels and maximising physician recall [19]. However, institutional restrictive policies at the hospital level moderate this effect [18]. Commercial detailing operates as an efficient sales driver but also creates informational asymmetries; AD interventions increasingly counterbalance this effect and demonstrate a 4.0% median improvement in guideline-concordant prescribing [4]. This contrast reflects a broader tension between commercial persuasion and evidence-based correction mechanisms, reinforcing the need for a multilevel explanatory framework.

Conceptual Framework: The Dynamics of Detailing

**Figure 2:**
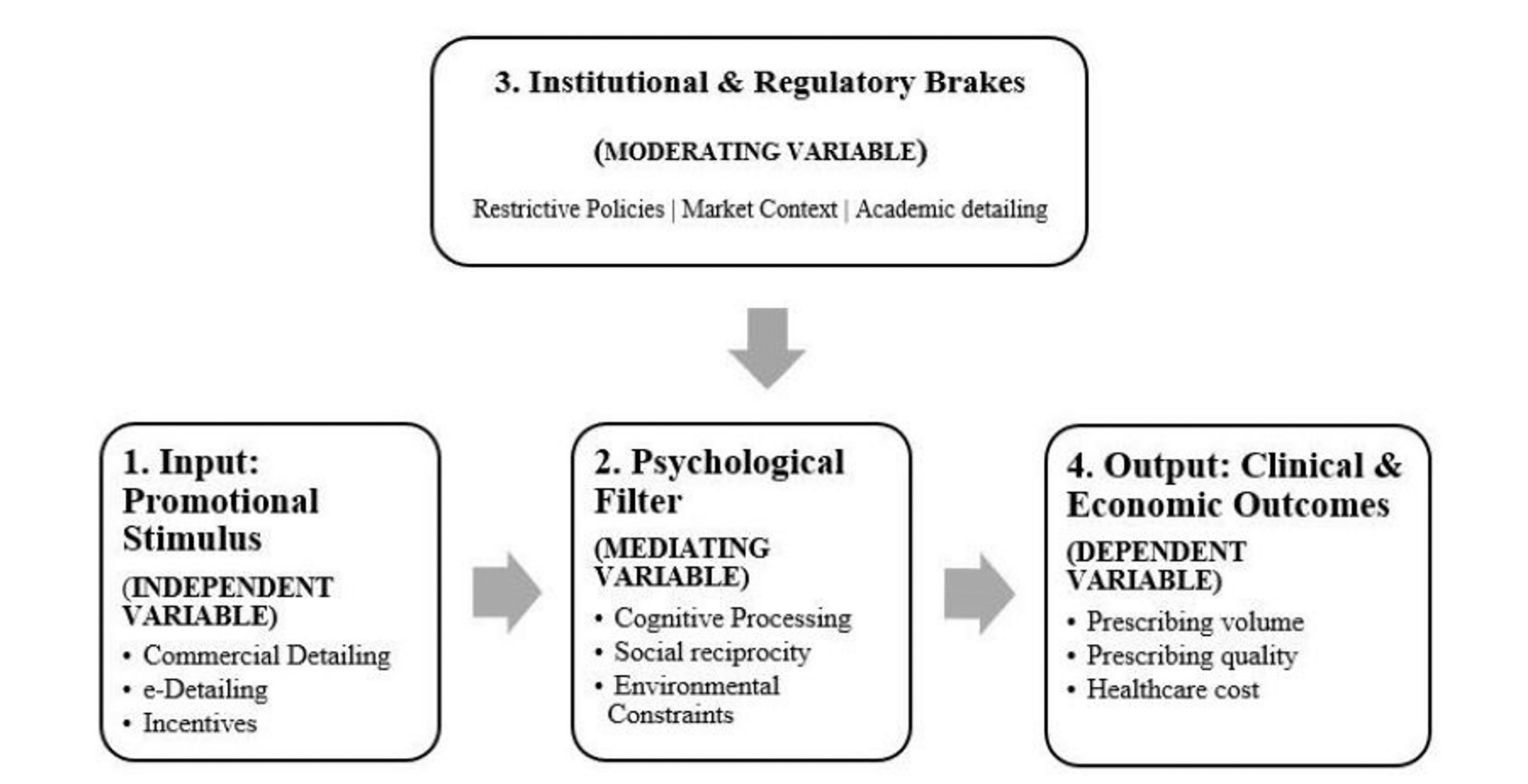
Conceptual Framework of Detailing Influence

This conceptual framework (Figure 2) integrates environmental stimuli, psychological mechanisms, and clinical outcomes to explain how pharmaceutical detailing transforms prescribing habits.

The process begins with promotional stimuli - the independent variables - where firms employ a marketing mix of face-to-face visits, drug samples, and digital e-detailing [3,7]. These inputs pass through a psychological "Filter", where cognitive processing via the ELM and the rule of social reciprocity mediate the doctor's response [17,2]. Acute environmental constraints, such as high patient volumes and abbreviated consultation times, further heighten physician reliance on industry-curated data [9]. Institutional and regulatory factors, including restrictive access policies at academic centres and the availability of generics, serve as critical moderators that can mitigate commercial influence [5,18]. Ultimately, these dynamics produce measurable dependent variables, specifically shifts in prescribing volume, therapeutic quality, and overall healthcare expenditure [19].

Stage 6: Consultation

Although the scoping review framework considers Stage 6 optional, this study included a limited consultation exercise to strengthen interpretation of the findings. The study obtained brief inputs from doctors and pharmaceutical professionals with relevant field experience. This step assessed whether the synthesized evidence aligns with current practice, without undertaking a formal qualitative investigation. The feedback confirmed overall consistency with the review findings.

Discussion

Our characterization of detailing as a sophisticated behavioural intervention rests on the consistent identification of psychological triggers within the included studies. High-quality evidence demonstrates that PSRs do not merely provide information but actively employ the ELM to leverage peripheral cognitive pathways [23]. For instance, evidence suggests that even low-value incentives-such as stationery or modest meals-activate a "norm of reciprocity," a social-psychological mechanism that compels physicians to return the favour through increased brand-name prescribing, often subconsciously [13]. Furthermore, researchers identify detailing as a tool that modifies the "Subjective Norm" component of the TPB, where the PSR’s presence validates the social acceptability of a specific clinical choice [18].

We use the term force multiplier to describe the operational efficiency detailing achieves in resource-constrained settings. In environments where Indian physicians manage high patient volumes with consultation times often averaging less than 10 minutes, the PSR functions as an essential informational shortcut [8]. The evidence indicates that commercial detailing "multiplies" the impact of marketing by providing condensed, high-utility summaries that influence the "extensive margin" - the initial decision to adopt a drug - which subsequent clinical experience then reinforces. Additionally, the transition to digital e-detailing acts as a force multiplier by overcoming geographical barriers and maintaining "mindshare" through persistent, multi-channel engagement that traditional face-to-face visits alone cannot achieve [20]. In resource-constrained environments with systemic educational gaps, detailing functions as a "Shadow CME" system. It acts as a "force multiplier" for the physician’s knowledge base, providing real-time technical support that formal institutional systems fail to deliver.

Beyond these traditional interpretations, commercial detailing functions as a de facto continuing medical education (CME) surrogate in regions with systemic educational gaps. The data reveals that in many instances, PSRs are the primary source of information regarding new drug-drug interactions and dosage adjustments, effectively filling a structural void in formal evidence-based knowledge translation [11]. By synthesizing these cognitive, operational, and structural dimensions, we maintain that our descriptors accurately reflect the complex, multi-layered influence detailing exerts on the modern clinical landscape.

The literature reveals a transformative shift in pharmaceutical detailing, moving from a mere sales tactic to a sophisticated instrument for behavioural change. While traditional commercial detailing functions as a marketing stimulus driven by material exchange [2,10], the rise of AD introduces a "Direct Social Marketing" paradigm [4]. This evolution suggests that the industry can ethically repurpose the persuasive power of detailing to serve public health by promoting guideline-concordant prescribing. The detailing significantly influences the "extensive margin" - the initiation of new prescriptions [5].

When looking at the synthesising stakeholder values, detailing accelerates the patient’s access to innovative therapies, and this transparency helps to prevent over-diagnosis [11]. From doctors' perspective, detailing preserves decision quality under high cognitive loads by substituting for exhaustive literature searches. When it comes to pharmaceutical industries the transitioning from "sales intermediaries" to "scientific communicators" allows firms to build trust-based relationship capital, ensuring sustainable ROI through ethical engagement [7].

We differentiate our review by adopting a more granular and mechanism-focused perspective. Specifically, we isolate commercial pharmaceutical detailing as a distinct, structured interpersonal intervention rather than treating it as one component within a broader set of industry interactions. This distinction allows us to examine detailing not only as a promotional activity but also as a behavioural stimulus operating through defined communication strategies and relational dynamics.

While foundational systematic reviews establish a consistent positive correlation between PSR visits and increased prescribing frequency, they often treat detailing as a singular, binary interaction. Our scoping review moves beyond this "interaction-outcome" paradigm by decomposing commercial detailing into specific operational mechanisms and theoretical variables. We differentiate our work through the following three unique contributions.

Mechanism-Specific Mapping

Unlike broad systematic reviews, we categorize detailing into discrete independent variables - such as cognitive framing, reciprocity triggers from low-value incentives, and regulatory constraints - to explain the "how" and "why" of prescribing shifts [10,20].

Hybrid Model of Detailing

We integrate contemporary evidence on the transition to hybrid "e-detailing" models. We uniquely contrast traditional face-to-face persistence with digital persistence, identifying how digital tools compensate for systemic time scarcity in high-velocity clinical environments where consultations may average less than 10 minutes [8].

Theoretical Delineation

We provide a comprehensive theoretical framework, contrasting Agency Theory and the TPB with the ELM. This allows us to map how PSRs utilize peripheral emotional processing and cognitive shortcuts to bypass rational clinical skepticism, even among specialized practitioners [17]. By synthesizing these multifaceted drivers, our review provides a conceptual model that bridges the gap between marketing theory and clinical practice, offering insights that traditional reviews focused solely on interaction frequency lack.

Theoretical Drivers and Future Gaps

Interlocking mechanisms like Agency Theory and the ELM explain the durability of detailings [17]. However, synthesis of the results reveals a critical "Digital Transition Gap." We do not yet fully understand how AI-driven e-detailing alters traditional reciprocity. There is no standardised methodology to measure the effectiveness of commercial detailing beyond sales metrics. Future research must explore this "hidden curriculum" of informal social norms to align commercial efficiency with clinical integrity.

The agency theory explains the information asymmetry observed across included studies, where physicians rely on PSRs to reduce search costs for drug information. This dependence introduces a moral hazard, as promotional inputs may influence clinical judgment, with evidence showing prioritisation of informational convenience over cost-effectiveness in prescribing decisions [13].

The TPB further clarifies that detailing shapes not only knowledge but also subjective norms, particularly through key opinion leader (KOL) endorsements. Empirical evidence from Indian and Jordanian settings demonstrates that such social cues significantly influence prescribing intentions, even in the absence of new clinical evidence [8,20].

The ELM provides a cognitive framework to interpret these effects, showing that physicians operating under time constraints and clinical uncertainty often rely on peripheral cues, such as promotional materials and reciprocity triggers, rather than central, evidence-based evaluation [23].

Integration of JBI appraisal findings further indicates that peripheral processing dominates in routine outpatient settings, whereas central processing is more evident in regulated academic environments. Collectively, this theory-driven synthesis advances the analysis from descriptive reporting to an explanatory framework that captures the behavioural and cognitive mechanisms underpinning commercial detailing [17].

Strengths and Limitations

This scoping review addresses a critical gap in pharmaceutical marketing research by mapping the variables of commercial detailing-a field that, unlike AD, lacks a standardised measurement framework. We enhanced the study's rigor by adhering to the PRISMA-ScR checklist and utilizing the JBI framework for data extraction and appraisal [26]. The review spans a comprehensive timeline (2014-2025), capturing both traditional face-to-face interactions and modern e-detailing trends, such as those governed by the UCPMP 2024. By integrating multiple theoretical lenses - Agency Theory, TPB, and ELM - the study provides a robust conceptual explanation for physician decision-making across diverse global contexts [17].

Despite its rigor, the review faces several limitations. The inclusion of non-medical prescribers, such as nurses and pharmacists, may introduce variability in how detailing influence is processed. Furthermore, the reliance on self-reported data introduces a risk of subjectivity bias; many clinicians exhibit an "illusion of invulnerability," believing they are immune to marketing while their peers are susceptible [2]. We also identified a persistent evidence gap regarding the lack of direct links between communication markers and objective pharmacy claims data. Finally, the focus on specific national directives, such as India’s UCPMP 2024 [24], may limit the global generalisability of some regulatory findings.

## Conclusions

The synthesis further reframes pharmaceutical detailing as a cognitive load outsourcing mechanism, where physicians operating under significant time and informational constraints selectively rely on curated inputs to manage decision complexity. Within this context, detailing does not function merely as information transfer but as a strategic intervention in the clinical attention economy, where PSRs compete to capture and sustain limited cognitive bandwidth. This dynamic elevates relational capital from a peripheral factor to a central determinant of influence, as repeated interactions accumulate trust and familiarity, thereby increasing prescribing responsiveness over time.

The analysis also highlights an “invisible influence” paradox, where the effectiveness of detailing intensifies when physicians perceive themselves as objective and unaffected, reinforcing the persistence of influence through subconscious cognitive pathways. Rather than passively reflecting informational gaps, detailing actively contributes to information asymmetry engineering, shaping not only what information physicians receive but also how they interpret clinical relevance within constrained environments.

From a systems perspective, detailing operates within a hybrid clinical intelligence architecture, where human interaction, commercial strategy, and digital augmentation converge to influence decision-making. Prescribing behaviour emerges from the interplay between behavioural ease (familiarity, repetition, relational trust) and behavioural friction (guideline constraints, institutional policies, and regulatory oversight). Early and repeated exposure to detailing further introduces path dependency, where initial prescribing patterns persist and stabilize over time, even in the presence of new evidence or regulatory interventions.

Regulatory frameworks extend beyond restrictive functions and act as informational signals that shape physician trust, interpretation, and engagement with pharmaceutical communication. This signalling function reconfigures influence pathways rather than eliminating them, thereby reinforcing the need to conceptualize detailing as a dynamic, adaptive system embedded within a policy-regulated communication ecosystem.

## Appendices

Search strategy

Keywords utilised: physician detailing, academic detailing, educational outreach visits, doctor, physician, prescriber, general practitioner & prescription decision; general filters English, time frame: 2014-2025

Pubmed Search string & Filters: (((((((physician detailing) OR (academic detailing)) OR (educational outreach visits)) AND (doctor)) OR (physician)) OR (prescriber)) OR (general practitioner)) AND (prescription decision); Filters: English, 1-1-2014 to 1-8-2025

Proquest Search string & Filters: ((physician detailing) OR (academic detailing) OR (educational outreach visits) AND doctor OR physician OR prescriber OR (general practitioner) AND (prescription decision)) AND (at.exact(("Feature" OR "Evidence Based Healthcare" OR "Literature Review" OR "Review") NOT ("Article" OR "Report" OR "Case Study" OR "Commentary" OR "General Information" OR "Editorial" OR "Correspondence" OR "Conference Proceeding" OR "News" OR "Letter to the Editor" OR "Undefined" OR "Correction/Retraction" OR "Interview" OR "Front Matter" OR "Table Of Contents" OR "Working Paper/Pre-Print" OR "Credit/Acknowledgement" OR "Obituary" OR "Audio/Video Clip" OR "Front Page/Cover Story" OR "Business Case" OR "Table of Contents" OR "Back Matter" OR "Bibliography" OR "Book" OR "Instructional Material/Guideline" OR "Poem" OR "Biography" OR "Dissertation/Thesis" OR "Speech/Lecture" OR "Conference" OR "Essay" OR "Statistics/Data Report" OR "Country Report" OR "Fiction" OR "Image/Photograph" OR "Market Research")) AND stype.exact(("Scholarly Journals" OR "Conference Papers & Proceedings") NOT ("Magazines" OR "Trade Journals" OR "Newspapers" OR "Other Sources" OR "Working Papers" OR "Reports" OR "Speeches & Presentations" OR "Books" OR "Encyclopedias & Reference Works")) AND la.exact("ENG") AND subt.exact(("studies" OR "patients" OR "physicians" OR "hospitals" OR "health care policy" OR "health care" OR "books" OR "medicine" OR "systematic review" OR "health care industry" OR "medical personnel" OR "health care expenditures" OR "literature reviews" OR "behavior" OR "clinical trials" OR "patient satisfaction" OR "students" OR "medical diagnosis" OR "professionals" OR "intervention" OR "attitudes") NOT ("health services" OR "public health" OR "decision making" OR "mortality" OR "research" OR "nurses" OR "population" OR "education" OR "chronic illnesses" OR "leadership" OR "ethics" OR "primary care" OR "collaboration" OR "employees" OR "older people" OR "disease" OR "economic theory" OR "health insurance" OR "quality of life" OR "diabetes" OR "trends" OR "innovations" OR "costs" OR "book reviews" OR "politics" OR "case studies" OR "emergency medical care" OR "clinical outcomes" OR "women health" OR "medicare" OR "women" OR "communication" OR "cost control" OR "surgery" OR "cancer" OR "analysis" OR "age" OR "artificial intelligence" OR "medical research" OR "cancer therapies" OR "pediatrics" OR "hypotheses" OR "families & family life" OR "medical treatment" OR "health economics" OR "society" OR "children & youth" OR "quality of care" OR "consumers" OR "pain" OR "employment" OR "mental health" OR "drug" OR "nursing" OR "risk factors" OR "information technology" OR "questionnaires" OR "epidemiology" OR "infections" OR "knowledge" OR "tumors" OR "statistical analysis" OR "economics" OR "economic models" OR "information systems" OR "metabolism" OR "success" OR "efficiency" OR "laboratories" OR "cardiovascular disease" OR "patient safety" OR "aging" OR "medicaid" OR "research methodology" OR "algorithms" OR "infectious diseases" OR "patient protection & affordable care act 2010-us" OR "clinical medicine" OR "health care delivery")) AND pd(20140101-20250801) AND PEER(yes))

Dimensions search string & filter: Criteria: '(physician detailing) OR (academic detailing) OR (educational outreach visits) AND doctor OR physician OR prescriber OR (general practitioner) AND (prescription decision)' in full data; Publication Year is 2025 or 2024 or 2023 or 2022 or 2021 or 2020 or 2019 or 2018 or 2017 or 2016 or 2015 or 2014; Fields of Research (ANZSRC 2020) is 42 Health Sciences or 4206 Public Health or 4203 Health Services and Systems or 32 Biomedical and Clinical Sciences or 5203 Clinical and Health Psychology or 3214 Pharmacology and Pharmaceutical Sciences or 3507 Strategy, Management and Organisational Behaviour or 3201 Cardiovascular Medicine and Haematology or 3215 Reproductive Medicine or 3204 Immunology or 3211 Oncology and Carcinogenesis or 3203 Dentistry or 5204 Cognitive and Computational Psychology or 4602 Artificial Intelligence; Sustainable Development Goals is 3 Good Health and Well Being or 4 Quality Education; Publication Type is Article or Edited Book or Chapter or Preprint or Proceeding; Source Title is The Journal of Rheumatology or Cochrane Database of Systematic Reviews or BMJ Open or PLOS ONE or Health and Social Care Delivery Research or SSRN Electronic Journal or JAMA Network Open or BMC Health Services Research or Critical Care or JAMA Internal Medicine or Journal of the American Pharmacists Association or Behavioral and Brain Sciences.

Googlescholar Search string & Filter: doctor OR physician OR detailing OR educational outreach visits physician OR prescriber OR general OR practitioner OR prescription OR decision "academic detailing"; English, Year:2014-2025.
